# Personality traits and social frailty in older adults: exploring the mediating effect of perceived loneliness

**DOI:** 10.3389/fpubh.2025.1637088

**Published:** 2025-08-29

**Authors:** Alberto Sardella, Vittorio Lenzo, Grazia Razza, Tiziana Maci, Samuele Russo, Dario Cannavò, Pasquale Caponnetto, Giorgio Basile, Maria C. Quattropani

**Affiliations:** ^1^Department of Educational Sciences, University of Catania, Catania, Italy; ^2^Unit of Psychogeriatrics, Department of Mental Health, Catania, Italy; ^3^Department of Biomedical and Dental Sciences and Morphofunctional Imaging, University of Messina, Messina, Italy

**Keywords:** older age, neuroticism, Social frailty, loneliness, clinical psychology

## Abstract

**Introduction:**

The relationship between personality traits, as defined by the Five Factor Model (FFM), and social frailty—according to the Gobbens model—represents a relatively novel area of research. Moreover, few studies have examined the link between personality and loneliness, a key determinant of social frailty, in older adults. This study aimed to explore the association between FFM personality traits and social frailty, and to assess whether perceived loneliness mediates this relationship.

**Methods:**

This cross-sectional observational study involved community-dwelling older adults attending a Geriatric Outpatients Clinic. Individuals aged ≥65 years were enrolled; those with diagnosed major neurocognitive disorders or psychiatric conditions were excluded. Personality traits were assessed using the Ten-Item Personality Inventory (TIPI), loneliness via the UCLA Loneliness Scale, and frailty through the Tilburg Frailty Indicator (TFI).

**Results:**

Data from 202 participants were analyzed (mean age: 74.45 ± 7.76 years; 57% female). Neuroticism was the only trait significantly associated with perceived loneliness (*r* = 0.190; *p* = 0.007). Perceived loneliness, in turn, was the sole variable significantly associated with social frailty (*r* = 0.526; *p* < 0.001). A mediation model (with age as covariate) revealed that loneliness fully mediated the relationship between Neuroticism and social frailty: the completely standardized indirect effect [β = 0.1017; 95% CI (0.0376, 0.1714)] confirms a moderate mediation effect.

**Conclusions:**

In older adults, higher levels of Neuroticism—reflecting a tendency toward negative affectivity—are linked to increased social frailty, primarily through the mediating role of perceived loneliness.

## Introduction

Personality traits are defined as relatively stable and enduring internal characteristics, which influence individual feelings, attitudes and behaviors. The Five Factor Model (FFM) provides an established representation of personality's structure. The FFM had originally assumed that most personality traits could be described in terms of five basic dimensions. Specifically, Neuroticism (i.e. tendency to experience negative emotions), Extraversion (i.e., being sociable and energetic), Openness (i.e., being curious and opened to new experiences), Agreeableness (i.e., being kind, affable, warm) and Conscientiousness (i.e., being organized, responsible, industrious and disciplined) (1). The contribution of personality traits on aging adaptation denotes a topic of broad interest. In this context, in line with the FFM, personality traits have been previously linked to several age-related outcomes, including cognitive decline (2), reduced autonomy in daily life (3), chronic conditions (4) as well as mortality (5).

Within aging trajectories, frailty denotes the most problematic manifestation of pathologic aging and it is defined as an increased vulnerability to stressors due to reduced homeostatic reserves; it is considered one of the most relevant age-related adverse outcomes (6). Two established models of frailty are the phenotype model (7) and the cumulative deficit model (8). The first defines frailty based on physical criteria (i.e., unintentional weight loss, self-reported exhaustion, slow gait speed, weak grip strength and decreased physical activity); the latter conceives frailty as resulting from the progressive accumulation of age-related deficits. A further model of frailty aimed at embracing a more comprehensive biopsychosocial perspective of the individual, combining physical, psychological and social domains of frailty; specifically, this model assumes that lifespan determinants have an effect on the occurrence of diseases, and they might jointly affect physical, psychological and social domains of frailty (9, 10). In the attempt of going beyond the merely physical definition of frailty, Gobbens et al (11) additionally acknowledged the psychological and social domains of frailty, which have been often neglected. With specific regard to the social domain of frailty, proposed for the first time by Gobbens et al. (10), it can be defined based on social indexes such as the condition of living alone, the poor quality of social relationships and the perceived low social support. It is known that older adults experience several age-related changes and losses, which may expose them to greater social isolation (12). In this perspective, social frailty has increasingly denoted a relevant public health issue, since it may expose older adults to a significant impairment of their social functioning, with negative consequences in terms of social engagement (13).

In the context of social closeness, loneliness is defined as that distressing experience occurring when social relationships are perceived quantitatively and qualitatively insufficient, compared to the desired or the expected ones (14). Loneliness has been broadly investigated as a relevant psychological factor in aging, since its significant effect on several age-related adverse outcomes, such as cognitive decline (15, 16), functional decline (17) and psychological wellbeing (18) as well as physical frailty (19).

The influence of dispositional features on frailty denotes a topic of interest and it has been previously investigated (20). Whereas the FFM of personality has been previously associated with physical (21) and cumulative (22) models of frailty in older adults, its association with social frailty, as conceptualized by the Gobbens model, to the best of our knowledge, still appears a novel topic. Furthermore, on the one hand, few studies have explored the association between personality traits and loneliness in older adults, nonetheless suggesting that some personality traits were differently associated with higher risk of loneliness in later life (23), on the other hand, loneliness can be considered a core determinant of social frailty (13).

In line with these premises, the purpose of the study was to investigate the association between FFM personality traits and social frailty; a further purpose was to investigate whether perceived loneliness might influence this association.

## Methods

### Participants

This observational and cross-sectional study was conducted among community-dwelling older adults attending a Geriatric Outpatients Clinic. All consecutive patients aged 65 years and older who attended the outpatient geriatric clinic for a scheduled routine appointment between March 2022 and January 2023 were invited to participate in the study. We excluded subjects with psychiatric disorders and/or neurocognitive disorders, including delirium, according to the DSM-5 diagnostic criteria (24). Specifically, despite cognitive functioning was not a variable of interest in the present study, all potential participants underwent cognitive screening using the Mini-Mental State Examination (MMSE) as part of the inclusion criteria. Only individuals with MMSE scores within the normal range (>24) were included in the sample. Written, freely given, informed consent to participate in the study was obtained from each participant.

### Measures

Preliminarily, in order to characterize the sample, sociodemographic information were collected (i.e. age, sex, years of educational level), as well as comorbidities (i.e. number of reported diseases). A trained clinical psychologist performed the psychological assessment.

In line with the purpose of the study, personality traits, perceived loneliness were measured as independent variables; social frailty was measured as dependent variable. Personality traits were evaluated through the Ten-Item Personality Inventory (TIPI) (25), in its Italian validated version (26). The inventory evaluates personality traits in line with the FFM (i.e., Extraversion, Agreeableness, Conscientiousness, Neuroticism, and Openness to Experience). The TIPI includes two items for each dimension—one positively worded and one negatively worded—resulting in a total of ten items. Subjects are asked to rate the extent to which they agree with each item using a 7-point Likert scale ranging from “Disagree strongly” to "Agree strongly; scores range from 1 to 7 per trait.

Perceived loneliness was measured by the UCLA Loneliness Scale (27), in its validated Italian version (28). This self-report scale comprises items that explore the following three dimensions of loneliness: relational connectedness, social connectedness and self-perceived isolation. Respondents are required to indicate how often they experience certain feelings related to loneliness on a 3-point Likert scale ranging as follows: “Hardly ever”, “Some of the time” and “Often.” The total score is obtained by summing the item responses, with higher scores indicating greater levels of loneliness.

Frailty was assessed through the Tilburg Frailty Indicator (TFI) (10), in its validated Italian version (29). The TFI is considered a valid and reliable instrument to detect frail individuals with a multidimensional approach, among older population (10, 11). Recognizing frailty as a complex construct, the TFI evaluates three interrelated domains: physical, psychological, and social frailty. The instrument consists of 15 items—eight addressing physical aspects such as mobility and fatigue, four relating to psychological issues like memory and mood, and three assessing social dimensions such as living situation and social support. Most items are answered dichotomously (yes/no), although some include a third “sometimes” option, which is recoded into a binary format for scoring purposes. The total frailty score is calculated by summing the responses across all items, yielding a score from 0 to 15. A score of 5 or higher typically indicates the presence of frailty. The Italian version of the TFI showed an acceptable internal consistency, with a Cronbach's α of 0.66 for the total TFI; values of internal consistency were 0.57, 0.51, and 0.36 for physical, psychological, and social domain, respectively (29). These properties are similar to those obtained for the original version of the TFI (10). The developers of the original TFI pointed out that increasing the number of items within each frailty domain might improve internal consistency; however, they opted to preserve the tool's brevity and ease of use, considering the lower internal consistency of individual domains an acceptable trade-off (10).

All participants fully completed the questionnaires and provided complete demographic and background information.

### Ethics statement

The study was conducted according to the guidelines of the Declaration of Helsinki, and approved by the Institutional Ethics Committee of the University Hospital of Messina (protocol number 23/19). Written informed consent was obtained from each subject involved in the study.

### Data analysis

Data were analyzed using *IBM SPSS.26* statistical software, and a non-parametric approach was chosen due to the results of the Shapiro-Wilk normality test, which indicated significant deviations from normality for all variables. The Spearman's coefficient were used for the correlation analysis; differences were analyzed by the Mann-Whitney test. Following the results from the correlation analysis, mediation analysis was performed through the PROCESS macro for SPSS; the “Bootstrap” method was used in order to perform such analysis and, in the present study, 5,000 bootstrap samples were used during mediation analysis, as suggested by Hayes (30). Age was used as covariate in the mediation analysis.

Values of *p* < 0.05 were considered significant. A *post-hoc* power analysis was performed through the *G* ^*^ Power software (version 3.1.9.6; Franz Faul, Edgar Erdfelder, Axel Buchner, Albert-Georg Lang, Germany), with a determined medium effect size of 0.18; a statistical power (1-β error probability) of 0.83 (critical *F* = 3.06) was reported.

## Results

The study included 202 community older adults (mean age 74.45 ± 7.76 years) with a female prevalence of 57.9%. Sociodemographic characteristics are summarized in Table 1.

**Table 1:** Main characteristics of the sample.

**Descriptives (*****N*** = **202)**		
**Variable**	**Mean**	**SD**
Age, years	74.45	7.76
Number of reported diseases	3.15	2.17
BADL	5.19	1.11
IADL men (*N* = 85)	5.27	1.57
IADL female (*N* = 117)	6.67	3.07
TFI total	4.56	2.67
TFI physical frailty	2.05	1.76
TFI psychological frailty	1.52	1.04
TFI social frailty	0.99	0.83
TIPI extraversion	7.63	3.32
TIPI agreableness	10.38	2.69
TIPI conscientiousness	10.76	2.64
TIPI nevroticism	7.27	3.29
TIPI openess	7.24	2.67
UCLA Loneliness Scale	4.47	1.71
**Prevalences (*****N*** = **202)**		
**Variable**	***N***	**%**
Female sex	117	57.9
***Education***		
0**–**4 years	9	4.5
Primary school diploma	41	20.3
Middle school diploma	40	19.8
High school diploma	66	32.7
Degree	42	20.8
Post-graduation	4	2
Frail subjects	101	50

SD, standard deviation; BADL, basic activities of daily living; IADL, instrumental activities of daily living; TFI, Tilburg Frailty Inventory; TIPI, ten item personality inventory. Male IADL ranges from 0 to 6; Female IADL ranges from 0 to 9. BADL scale and IADL scale are expressed as number of maintained functions. Note: a subject is considered frail if the total TFI score is ≥ 5.

Female participants reported significantly higher levels of social frailty then men (1.19 ± 0.81 vs 0.71 ± 0.78; *p* < 0.001), and marginally significant higher levels of Neuroticism then men (7.62 ± 2.94 vs 6.79 ± 3.67; *p* = 0.053). Subjects classified as frail (TFI total score ≥ 5) reported significantly higher levels of Neuroticism (8.29 ± 2.88) then those not frail (6.25 ± 3.37; *p* < 0.001) and lower levels of Openess (6.64 ± 2.70) then those not frail (7.83 ± 2.51; *p* = 0.001). Frail subjects reported also higher levels of perceived loneliness (5.08 ± 1.8) compared to those not frail (3.85 ± 1.85; *p* < 0.001).

### Correlation analysis

Among all personality traits, only Neuroticism showed a significant correlation with perceived loneliness (*r* = 0.190; *p* = 0.007).

Physical frailty was positively correlated with loneliness (*r* = 0.238; *p* = 0.001) and with Neuroticism (*r* = 0.200; *p* =0.04), and negatively correlated with Conscientiousness (*r* = −0.224) and with Openess (*r* = −0.270; both *p* values < 0.001). Psychological frailty was positively correlated with loneliness (*r*= 0.313) and with Neuroticism (*r* = 0.464; both *p* values < 0.001), and negatively correlated with Extraversion (*r* = −0.142; *p* = 0.04), with Conscientiousness (*r* = −0.150; *p* = 0.03) and with Openess (*r* = −0.170; *p* = 0.03).

Perceived loneliness was the only psychological factor correlated with social frailty (*r* = 0.526; *p* < 0.001).

### Mediation analysis

Following the correlation analysis, a mediation model was tested, including social frailty as the dependent variable, in order to test whether perceived loneliness mediates the association between personality traits and social frailty (Table 2). The model controlled for age, which was included as a covariate in both the mediator (*m*) and outcome (*y*) equations. Mediation analyses were conducted for each of the FFM personality traits. Only neuroticism yielded a significant indirect effect through perceived loneliness on social frailty. Age showed no significant effect to loneliness (β = 0.05; *p* = 0.42) nor to social frailty (β = −0.0067; *p* = 0.91).

**Table 2:** Mediation analysis: total, direct and indirect (via loneliness) effects of nevroticism on Social frailty.

	**Effect**	**SE**	***t***	***p***	**LCI**	**UCI**
Total effect	0.0368	0.0180	2.0470	0.0420	0.0014	0.0723
Direct effect	0.0109	0.0162	0.6739	0.5011	−0.0211	0.0430
**Mediation by perceived loneliness**						
	**Effect**	**BootSE**	**BootLCI**	**BootUCI**		
Indirect effect	0.0259	0.0091	0.0096	0.0455		
Standardized indirect effect	0.1017	0.0343	0.0376	0.1714		

SE, standard error; LCI, lower 95% confidence intervals; UCI, upper 95% confidence intervals; Boot, bootstrap samples (=5,000). Age was included as covariate in the model.

**Figure 1:**
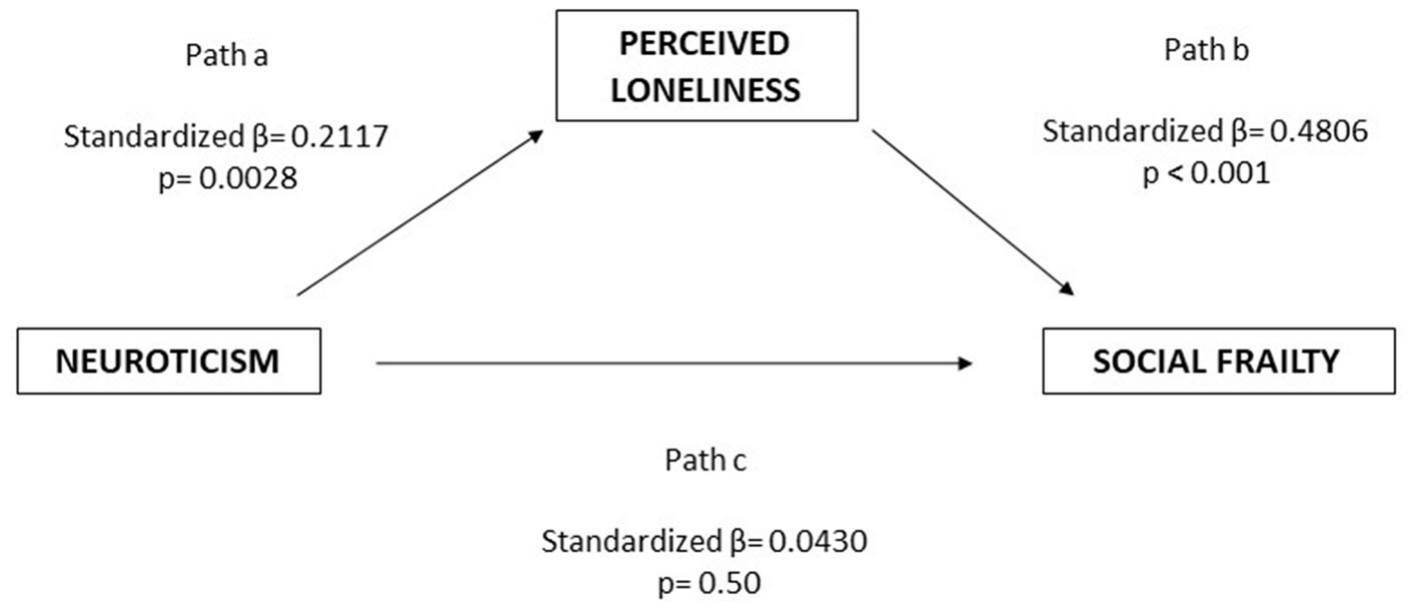
Path analysis between Neuroticism, perceived loneliness and social frailty. Perceived loneliness fully mediated the association between Neuroticism and social frailty.

The total effect of neuroticism on social frailty was significant (*B* = 0.0368, *p* = 0.042), suggesting that higher levels of neuroticism are associated with greater social frailty. However, the direct effect became non-significant when loneliness was included as a mediator (*B* = 0.0109, *p* = 0.501), indicating that the relationship is fully mediated by perceived loneliness. The indirect effect through loneliness was significant [*B* = 0.0259; 95% CI (0.0096, 0.0455)], and the completely standardized indirect effect [β = 0.1017; 95% CI (0.0376, 0.1714)] confirms a moderate mediation effect (Figure 1).

## Discussion

The aim of this study was to examine the contribution of personality traits, as well as loneliness, to social frailty, as conceptualized by Gobbens' multidimensional model of frailty. Social frailty has been recognized as a risk factor for healthy aging, as it is significantly associated with the loss or reduction of social resources and activities, which are essential to meeting individual social needs (31, 32).

Regarding sex differences, the present study found that women exhibited higher levels of social frailty compared to men. This result is consistent with the higher prevalence of frailty among older females (11, 33), and aligns with a recent meta-analysis reporting that the overall prevalence of multidimensional frailty, as measured by the TFI, was higher in women than in men (34).

In the context of social frailty, it is worth emphasizing the role of loneliness—defined as the subjective feeling of social disconnection—which constitutes a core determinant of social frailty (13). As expected, our findings showed that perceived loneliness was significantly associated with TFI social frailty. The relationship between loneliness and social frailty is supported by the well-established notion that older adults are more vulnerable to experiencing loneliness, due to the decline of various indicators of social well-being (e.g., frequency of social contacts and activities, network size) in later life (35). These changes may shape potential pathways toward social frailty.

Another noteworthy finding was that higher levels of perceived loneliness were also associated with higher levels of Neuroticism. In line with previous research in older adults (36), this association may be interpreted by the negative influence of the neurotic trait on social relationships. Individuals high in Neuroticism tend to experience negative emotions, which can intensify feelings of loneliness. Furthermore, it can be hypothesized that Neuroticism may contribute to a biased—specifically negative—interpretation of social situations, thereby exacerbating perceived loneliness (37). This observation may also be framed within the broader assumption that emotional regulation and specific personality traits are mutually linked (38).

As outlined in our premises, the exploration of the relationship between the FFM of personality and Gobbens' multidimensional model of frailty remains relatively novel. To the best of our knowledge, this is the first study to investigate the association between personality traits and TFI social frailty. Although no direct associations between FFM personality traits and social frailty were found, our results revealed that perceived loneliness fully mediated the relationship between Neuroticism and social frailty. This suggests that, in the presence of a neurotic personality trait, the key to understanding vulnerability to social frailty may lie in the subjective discrepancy between one's ideal socio-emotional needs and the perceived fulfillment of those needs.

From a theoretical perspective, loneliness serves as a warning signal of unmet needs (39). While not all experiences of loneliness are inherently negative (35), they may become problematic when accompanied by high levels of Neuroticism, as previously discussed. In this light, social relationships require a significant investment of time and energy—an investment older adults often view as essential to maintaining their social functioning (40). However, when these expectations go unmet, older individuals—already prone to negative emotions—may perceive themselves as lonely, leading to reduced motivation for social engagement and a progressive withdrawal into social frailty.

This study suggests that perceived loneliness functions as a crucial link between the tendency to experience negative affectivity (i.e., Neuroticism) and social frailty in older adults. However, some limitations must be acknowledged, such as the cross-sectional design, which prevents the exploration of longitudinal trajectories. Moreover, although our results are supported by an acceptable *post-hoc* power analysis, future studies with larger samples are needed to confirm and expand upon these findings, also by considering the potential contribution of additional variables that may directly or indirectly influence the model under investigation. Indeed, although the present findings suggest a meaningful link between personality traits—particularly Neuroticism—and social frailty via perceived loneliness, we did not deepen the practical aspects of implementing these findings in clinical settings. The process of translating this evidence into real-world interventions remains complex and untested. Implementing personality-based assessments and tailoring interventions to individual personality profiles on a large scale would require additional research to explore feasibility, cost-effectiveness, and clinical effectiveness in older populations at risk of frailty; future studies should thus focus on these unmet needs. Ultimately, as the MMSE was used exclusively to exclude individuals with cognitive impairment, this approach ensured a more homogeneous sample in terms of global cognitive functioning. As a result, any associations observed in the study are unlikely to be confounded by underlying cognitive deficits. However, it should be noted that this choice may limit the generalizability of the findings to cognitively intact older adults, and does not allow exploration of how varying levels of cognitive functioning might interact with the variables under investigation.

Nonetheless, this study offers novel insights that may be framed within the recently proposed and compelling concept of Affective Reserve, defined as the capacity to regulate affective states and dispositions, and which helps explain individual differences in the ability to cope with stressful or adverse life events (41). Ultimately, it is increasingly important to recognize social frailty, which is often overlooked or only partially assessed. Doing so would support the development of tailored and effective clinical psychological interventions for older adults. From a clinical standpoint, interventions targeting loneliness may help mitigate the adverse effects of Neuroticism, thereby fostering greater social resilience in the aging population.

## Data Availability

The raw data supporting the conclusions of this article will be made available by the authors, without undue reservation.
